# Drug-Induced Lung Injury With Drug Eruption Due to Apalutamide Followed by Switch to Bicalutamide: A Case Report

**DOI:** 10.7759/cureus.91764

**Published:** 2025-09-07

**Authors:** Nao Tanaka, Kanayuki Kitahara, Yumiko Kusunoki, Tomiko Sunaga

**Affiliations:** 1 Department of Hospital Pharmaceutics, School of Pharmacy, Showa Medical University, Tokyo, JPN; 2 Department of Pharmacy, Showa Medical University Fujigaoka Hospital, Kanagawa, JPN; 3 Department of Pharmacy, Kameda Medical Center, Chiba, JPN; 4 Department of Pharmacy, Showa Medical University Northern Yokohama Hospital, Kanagawa, JPN; 5 Division of Applied Pharmaceutical Education and Research, Hoshi University, Tokyo, JPN

**Keywords:** androgen receptor inhibitors, apalutamide, bicalutamide, drug-eruption, drug-induced lung injury, prostate cancer (pca), prostate cancer therapy

## Abstract

Androgen receptor inhibitors (ARIs) are the first-line treatment for castration-resistant prostate cancer. However, there have been limited reports on drug-induced lung injury (DILD) and other serious adverse events. We report the case of an 85-year-old Japanese man who was diagnosed with prostate cancer and initiated treatment with apalutamide. Approximately two months later, he developed DILD accompanied by a drug eruption. After recovery, the patient was switched to bicalutamide, and the drug eruption recurred; however, DILD did not develop. We suspected cross-reactivity in the drug eruption but not in the interstitial lung injury. This case suggests that medical practitioners should be careful concerning the development of drug eruption and DILD when switching from one ARI to another when DILD develops in an ARI.

## Introduction

Androgen receptor inhibitors (ARIs) are widely used in the treatment of prostate cancer because they are as effective as castration and better tolerated than cytotoxic agents [1]. Bicalutamide is the first-generation ARI for castration-resistant prostate cancer (CRPC) cases, while apalutamide represents the second generation. According to the National Comprehensive Cancer Network, apalutamide is the first-choice treatment for CRPC [1]. Although generally well-tolerated, serious adverse events, including drug-induced lung injury (DILD), have been reported with their use [2]. However, there are no reports on the possibility of switching to other ARIs as alternatives to apalutamide in the event of adverse reactions.

In this study, we report the case of a patient who developed DILD and a drug eruption from apalutamide. The patient was subsequently switched to bicalutamide and did not develop DILD; however, a drug eruption with suspected cross-reactivity occurred.

## Case presentation

An 85-year-old Japanese man was diagnosed with prostate cancer (T3aN1M1b) after a biopsy performed five months prior to admission. Written informed consent was obtained from the patient.

**Figure 1:**
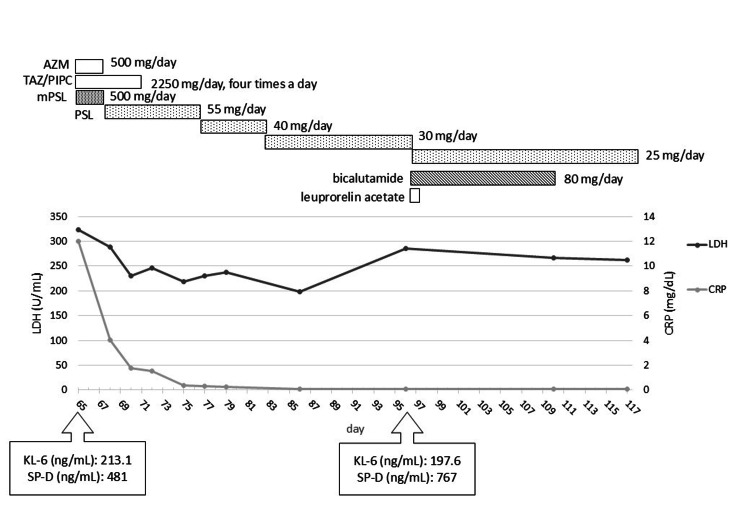
Clinical course of the case The image is created by the author. The patient's medication history and clinical course are presented in the figure. The graphs show the patient's lactate dehydrogenase (LDH), C-reactive protein (CRP), Krebs von den Lungen-6 (KL-6), and surfactant protein-D (SP-D) from the day of admission.

The patient had a myocardial infarction, hypertension, and diabetes mellitus. He had no history of allergies, smoked 40 cigarettes per day for 32 years (quitting in his 50s), and did not consume alcohol. His medications included aspirin enteric tablets (100 mg/day), carvedilol (10 mg/day), azelnidipine (8 mg/day), candesartan (8 mg/day), esomeprazole (20 mg/day), rosuvastatin (2.5 mg/day), alogliptin (25 mg/day), magnesium oxide powder (1 g/day), rebamipide (100 mg/day), Tsumura Daikenchu-tang for abdominal discomfort, etizolam (0.25 mg) for insomnia, mepenzolate (7.5 mg) for abdominal pain, and loxoprofen tape (50 mg) for pain relief. The patient's medication history and clinical course are presented in Figure 1.

**Figure 2:**
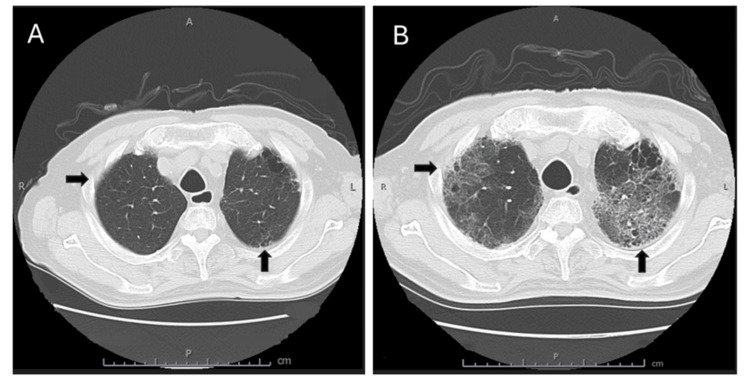
CT images of this case taken before starting apalutamide treatment and at the time of hospitalization (day 65). CT: Computed tomography A. CT images taken before starting apalutamide treatment did not show NSIP. B. CT images taken at the time of admission (day 65) suggested NSIP.

Degarelix acetate (240 mg/dose) was administered three months prior to admission. Injectable leuprorelin acetate (11.25 mg/dose) and apalutamide (240 mg/day) were administered two months prior to admission (day one). On day 58, the patient reported fatigue and erythema of the trunk and extremities during an outpatient visit. The physician suspected a rash due to apalutamide, leading to the discontinuation of apalutamide and prescription of olopatadine at 10 mg/day. On day 65, the patient presented with dyspnea and fatigue upon exertion, and his oxygen saturation (SpO_2_) was 95% on room air. Chest radiography revealed extensive ground-glass opacities in both lung fields, and a computed tomography (CT) examination suggested non-specific interstitial pneumonia (NSIP) (Figure 2B). The patient was urgently admitted to the hospital.

The patient's vital signs on admission were as follows: temperature, 37.1°C; blood pressure, 71/48 mmHg; heart rate, 51/min; and respiratory rate, 15/min. SpO_2_ was 89%, prompting the initiation of oxygen therapy at 1 L/min. Concomitant medications were continued post-admission. CT imaging revealed pleural predominance in the entire right lung area and multiple abrasions and reticular shadows in the left upper lobe and tongue area, suggesting interstitial pneumonia in the NSIP pattern (Figure 2B). The patient also presented with dyspnea on exertion and high levels of KL-6 (481 ng/mL), surfactant protein-D (213 ng/mL), and ferritin (126.6 ng/mL). Steroid pulse therapy (methylprednisolone 500 mg for 3 days) was initiated on day 65. A drug-induced lymphocyte stimulation test (DLST) was conducted on the same day. Tazobactam piperacillin hydrate (2,250 mg/day, four times a day) and Azithromycin hydrate (500 mg/day) were also initiated on day 65. The COVID-19 antigen, COVID-19 polymerase chain reaction, urinary pneumococcal antigen, influenza AB antigen, urinary Legionella, and β-D glucan 7.2 test results were negative. To rule out collagen disease, the patient was tested for various collagen disease markers, including antinuclear antibody, antinuclear antibody, SPECKLED, cytoplasmic antineutrophil cytoplasmic autoantibody, anti-myeloperoxidase antibody, anti-aminoacyl tRNA synthetase antibody, anti-Sjögren's-syndrome-related antigen A antibody, and non-specific IgE. The DLST results for apalutamide were negative.

On day 66, the physician prescribed betamethasone valerate ointment and heparin-like ointment for the drug eruption; olopatadine was discontinued, and the patient was monitored. Lupatadine fumarate (10 mg/day) was additionally prescribed on day 70 because of increasing itching. The drug eruption crusted and improved by day 83. Post-therapy for NSIP was initiated with prednisolone (PSL) 55 mg (approximately 1 mg/kg) from day 68, which gradually tapered off. With steroid therapy, the symptoms of dyspnea were alleviated and the skin symptoms improved; therefore, the dose was gradually reduced to 30 mg/day, and the patient was discharged from the hospital on day 86.

On day 96, the patient's prostate-specific antigen (PSA) level was elevated at 1.24 ng/mL, and injectable leuprorelin acetate and bicalutamide at 80 mg/day were administered. On day 110, during an outpatient visit, erythematous, non-touchable, diffuse plaques were observed on the trunk and extremities, consistent with a drug eruption. As there were no symptoms on the face, the drug eruption was attributed to bicalutamide rather than photosensitivity, leading to the discontinuation of bicalutamide. For drug eruption, a combination of epinastine hydrochloride tablets (20 mg/day), betamethasone butyrate propionate ointment, and heparin-like ointment was prescribed. A chest X-ray examination showed no abnormalities, and SpO2 remained stable at 98%. The patient's skin symptoms improved, and the PSL dose gradually decreased.

## Discussion

In this case, the patient developed an interstitial lung injury (ILI) and a drug eruption due to apalutamide. After switching to bicalutamide, a drug eruption occurred within two weeks, suggesting a suspected cross-reaction. However, no cross-reactivity or ILI onset was observed. Following the discontinuation of apalutamide, the PSA level increased, prompting a switch to bicalutamide. Examination results indicated that the interstitial lung disease in this patient was unlikely to be caused by infection or collagen disease. The patient continued receiving all medications except apalutamide after admission, and the ILI symptoms did not exacerbate, suggesting that these medications were unlikely to have contributed to these symptoms.

Adverse events associated with apalutamide and bicalutamide included two cases of apalutamide-induced interstitial lung injury (ILI), one diagnosed and treated 3-4 months after initiating apalutamide [2]. Nawa et al. also reported apalutamide-induced ILI [3], including a case where rash and ILI occurred simultaneously, similar to the present case. The Naranjo score for apalutamide-induced ILI was 8 points. Maeda et al. reported bicalutamide-induced ILI [4], although this did not occur in the present patient.

Therefore, we concluded that both apalutamide and bicalutamide induced drug eruptions and ILI. In the present case, apalutamide caused both a drug eruption and ILI, followed by a drug eruption caused by bicalutamide, which could be considered a cross-reaction. Although a skin biopsy was not performed, the skin rash was judged to be drug-induced.

One of the factors that may have contributed to cross-reactivity in drug eruptions is the similarity of the structural formulas of the two drugs. Apalutamide’s structure includes cyanopyridine, which could react with cysteine residues of serum proteins to form haptens [5]. Although bicalutamide does not contain cyanopyridine, it does have cyano and trifluoromethyl groups, which are also present in apalutamide. This structural similarity may be related to the observed cross-reactivity.

However, the absence of cross-reactivity in the ILI could be attributed to steroid treatment. Although steroids are not universally proven effective against DILD [6], their anti-inflammatory and immunosuppressive effects may have masked ILI symptoms in this case.

Although the pathogenesis and pathophysiology of DILD are not fully understood, two primary mechanisms are generally considered: direct (drug accumulation due to the drug itself, drug interaction with other drugs, or abnormal metabolism) and indirect cytotoxic effects, such as allergy. In the former, the lung cells themselves are damaged by cytotoxic drugs, such as antineoplastic drugs, and the onset of the disease is often chronic (weeks to years). In contrast, the latter is thought to be caused by an immune response to the drug, where the drug acquires antigenic properties as a hapten, potentially leading to lung injury in many cases. In this case, the disease developed within a relatively short period (1-2 weeks) after drug use. Recently, a combination of these two approaches was reported [7].

In this case, the disease onset was chronic, lasting a few months, with no recurrence upon switching to bicalutamide, which shares a similar structural formula. This suggests that the pulmonary damage caused by apalutamide may have resulted from a direct mechanism. In addition, our findings suggest that allergic adverse events, such as erythematous papules, may stem from cross-reactivity between drugs with similar structural formulas and specific functional groups.

## Conclusions

Drug-induced ILI can be caused by two mechanisms: allergies and direct injury. Androgen receptor antagonist-induced ILI may be caused by a direct injury mechanism. Healthcare providers should exercise caution regarding the development of drug eruptions and DILD when managing patients who develop DILD while receiving one ARI and subsequently switch to another.
